# A high‐resolution physiological time‐series uncovers strong but variable seasonal acclimation of copepod thermal limits

**DOI:** 10.1002/ecy.70564

**Published:** 2026-10-26

**Authors:** Matthew Sasaki, Allison Rodger, Chanchal Saratkar, Jamie Cull‐Host, Sydney Sharp, Melissa Pespeni

**Affiliations:** ^1^ Department of Biology University of Vermont Burlington Vermont USA; ^2^ Department of Biological Sciences University of Massachusetts Lowell Lowell Massachusetts USA

**Keywords:** acclimation, community, copepod, CTmax, plasticity, seasonality, thermal limits

## Abstract

How a community responds to warming depends on both intraspecific variation in thermal limits and variation in acclimation capacity across community members. These factors are often overlooked, however, leading to uncertainties about how climate change affects biodiversity. In temperate regions, communities are exposed to large seasonal temperature fluctuations, providing an opportunity to examine community‐wide effects of acclimation under natural conditions. Generating a weekly physiological time series over a one‐year period, we examined seasonal variation in upper thermal limits for the entire community of calanoid copepods in Lake Champlain, a large and highly seasonal lake in North America. Thermal limits varied substantially between species, with winter‐dominant species having lower thermal limits than summer‐dominant species. Our high‐resolution temporal data also revealed substantial but variable acclimation capacities across community members. Hindcasted vulnerability estimates highlight the importance of species‐specific estimates of acclimation capacity for robust predictions about the impacts of warming. Over the course of our collections, we also recorded two novel species occurrences in Lake Champlain, both of which were notable for their high upper thermal limits. Our results highlight the importance of acclimation in shaping population responses to temperature variation under natural conditions.

## INTRODUCTION

Predicting the effects of climate change on biodiversity is a pressing challenge. Faced with rapid warming across lakes and standing waters in many regions (Kraemer et al., 2021; Woolway et al., 2020), freshwater aquatic organisms may be particularly vulnerable to anthropogenic changes (Sayer et al., 2025). As temperatures increase, there have been widespread observations of shifts in community composition, population abundances, and range distributions across many taxa (Khaliq et al., 2024). Diverse physiological and ecological mechanisms may underlie these changes, but variation in thermal performance traits (and upper thermal limits in particular) across taxa has received particular attention (Calosi et al., 2007; Roeder et al., 2021). As a result, spatial patterns in thermal limits are often used to predict patterns in vulnerability to climate change (Pinsky et al., 2019).

Many of these studies assume, however, that thermal limits are invariant within species. A growing body of literature shows this is clearly not the case. Genetic variation across populations (Sasaki et al., 2022) and physiological plasticity (Morley et al., 2019) can produce substantial intraspecific variation in thermal limits that is important to consider for robust predictions about the effects of climate change on biodiversity (Jacob & Legrand, 2021). Population responses to temperature seasonality can also entail substantial and rapid shifts in organismal performance (Garton et al., 1990; Hahn & Brennan, 2024; Harada et al., 2018; Huey & Buckley, 2022; Leclair et al., 2020). Importantly, both adaptation and plasticity may play important roles in shaping how populations of short‐lived taxa cope with seasonally varying temperatures (Bergland et al., 2014; Elmer et al., 2025; Hahn & Brennan, 2024; Rudman et al., 2022; Sasaki & Dam, 2020; Ueno et al., 2023). This temporal variation in thermal limits may mediate vulnerability to both acute and long‐term warming events (Dowd & Denny, 2020). Understanding the extent to which populations rely on these different mechanisms to persist in variable environments can provide key insights into how long‐term climatic changes may affect biodiversity.

While highly controlled laboratory experiments can provide crucial mechanistic insight into seasonal variation in thermal limits (i.e., determining whether phenotypic variation is genetic or plastic in origin), assessments under more realistic conditions are also needed. The idealized, stable conditions and experimental designs typically used in laboratory environments may, for example, mask how other factors like food limitation, behavioral thermoregulation, photoperiod, and humidity influence thermal limits in natural populations (Ueno et al., 2023). Laboratory estimates of the effects of plasticity are also subject to a number of potential methodological artifacts (Rohr et al., 2018). Given the important (but uncertain) role plasticity may play in mediating vulnerability to warming (Gunderson & Stillman, 2015; Morley et al., 2019; Seebacher et al., 2015), we need to complement laboratory studies with a better understanding of potential constraints on thermal limits and the effects of plasticity under realistic environmental conditions. These constraints (including natural seasonal variation in organismal condition) may be particularly important for contextualizing environmental stress (Dowd & Denny, 2020) and grounding predictions about how interactions between plasticity and adaptation may shape population responses in a changing climate (de Lisle & Rowe, 2023; Diamond & Martin, 2016).

Further, while describing seasonal variation within a single species can provide useful insights, variation in both thermal limits and acclimation capacity across community members will likely play a major role in determining “winners and losers” in a changing climate (Roeder et al., 2021; Somero, 2010). These dynamics are important to understand in order to predict how climate change may alter ecological dynamics at a larger scale. Despite this, very few studies have examined in situ seasonal patterns in thermal limits across community members, limiting our ability to compare the effects of acclimation. Those that have (Bujan et al., 2020; García et al., 2024; Haque et al., 2025; Hopkin et al., 2006; Houghton & Shoup, 2014; Hu & Appel, 2004; Oliveira et al., 2021; Redana et al., 2024) often uncover different magnitudes of variation in thermal limits across species, which suggests variable acclimation capacity across community members. These studies have, however, tended to focus on a small number of community members, just a portion of the annual temperature cycle, or have covered the entire year with fairly coarse (i.e., monthly) sampling intervals. This limits insights into patterns of acclimation under natural conditions and how winter‐active species respond to warming (Dinh et al., 2023; Hampton et al., 2017; Pu et al., 2025). This has also resulted in a limited understanding of how variation in physiological thermal limits affects ecological patterns like seasonal community turnover, long‐term shifts in community composition, and changes in phenologies observed in various aquatic systems (Corona et al., 2024; Khaliq et al., 2024). A high temporal resolution, community‐wide examination of acclimation under natural conditions, spanning the full annual temperature cycle, may help to establish a mechanistic foundation for understanding population responses to changing seasonality (Hernández‐Carrasco et al., 2025; Williams et al., 2017).

Planktonic copepods are a useful model system for studying variation in thermal limits and acclimation capacity. These taxa are widespread, diverse, and abundant throughout the year in many freshwater systems. This group is also ecologically important, as grazers of phytoplankton and microzooplankton and prey for early life stages of many fish species. While historically thermal limits and acclimation capacity in freshwater copepods have been largely overlooked (Sasaki & Dam, 2021), there has been renewed interest in these systems (Bonadonna et al., 2025; Nowakowski & Sługocki, 2024).

Altogether, there is both a distinct need to better understand patterns in freshwater copepod thermal limits and an opportunity to use this system to extend patterns observed in laboratory experiments to natural communities. Here, we examine seasonal patterns in upper thermal limits of the entire calanoid copepod community in Lake Champlain, a large, highly seasonal lake in temperate North America. Sampling at approximately weekly intervals over a one‐year period, we generated a physiological time series to examine across‐ and within‐species variation in thermal limits as well as species‐specific acclimation capacities under natural conditions. Previous work suggests that thermal limits vary across species corresponding to the thermal environment experienced and that acclimation capacity is highest in species with the lowest thermal limits (i.e., that there is a trade‐off between upper thermal limits and acclimation capacity; Sasaki & Dam, 2021; Stillman, 2003). As such, we hypothesize that summer‐dominant species will have higher thermal limits but lower acclimation capacities than winter‐dominant species. Finally, combining our data with a continuous surface temperature record for Lake Champlain, we use a hindcasting approach to quantify the importance of considering species‐specific acclimation capacities when predicting vulnerability to warming.

## METHODS

### Field collections and environmental data

Plankton were collected within the top 3 m of Lake Champlain (coordinates: 44.481149, −73.225451) with a combination of horizontal and vertical tows of a 500‐μm mesh plankton net. These collections were not intended for quantitative measurement of abundance, so the precise volume of water filtered was not tracked. Surface water temperature was measured at the time of collection using a handheld thermometer. Plankton were transferred to an insulated flask and immediately transported to the laboratory at the University of Vermont, where the tow contents were divided among several 500‐mL beakers and placed in a benchtop incubator (IVYX Scientific) set to the temperature recorded at the time of collection. The United States Geological Survey (USGS) maintains a continuous temperature record at a nearby site (~650 m from our sampling site; coordinates: 44.47616017, −73.2215166), which allowed us to examine more fine‐scale patterns across the entire collection period for this project (May 25, 2023 to May 20, 2024). Temperatures recorded at the time of collection closely matched the maximum daily temperatures from the continuous temperature record (Appendix S1: Figure S1).

### Trait measurements

Thermal limits were measured as the critical thermal maximum (CTmax), the temperature at which an individual no longer responded to stimuli (i.e., onset of heat coma), using a previously developed method (Sasaki et al., 2023). The experimental setup includes a reservoir containing a heating element and two aquarium pumps, a plexiglass water bath, and a continuous temperature recorder. One aquarium pump circulates water within the reservoir, while the other pumps water into the water bath. The water bath contains thirteen 50 mL flat‐bottom glass vials, each with 10 mL of bottled spring water. Small temperature sensors connected to an Arduino logger were placed into three of these vials. Temperature was recorded every 5 s during the experimental assays.

To begin each CTmax assay, the water in the reservoir was adjusted to match the collection temperature using either the heating element or by adding ice. While variation in starting temperature can affect CTmax values (Terblanche et al., 2007), using a consistent start temperature across the duration of this project was not feasible, given the large seasonal range of temperatures observed in Lake Champlain (from ~0 to 25°C during our sampling period). The various species collected differed in their experienced temperature range, with only minimal overlap across the species at approximately 10°C. Were we to use this common start temperature, it would have (1) introduced potential confounding effects of large temperature changes immediately preceding CTmax assays as organisms were moved from field temperatures to the standardized start temperature, and (2) greatly exacerbated the difference in experiment duration across the seasons (large increase in experiment duration for the summer species, but a reduction for the winter species). We also note that the effect of starting temperature can be variable, even across populations of the same species (Cicchino et al., 2023). Finally, variation in start temperatures appears to have the largest effects when start temperatures are near or above the critical temperature (Faber et al., 2026); it is unlikely that collection temperatures were consistently above the critical temperature for any given species in our data set, thus likely minimizing the effect of variation in start temperature. In cases like this, field‐based starting temperatures are commonly used (Shah et al., 2017; Terblanche & Hoffmann, 2020), although this can complicate the inclusion of these measurements in syntheses or in cross‐study comparisons.

When the water bath was holding steady at the collection temperature, calanoid copepods were sorted from the bulk plankton to tow contents and placed individually into the remaining 10 vials. After all vials were filled, copepods were given a 15‐min resting period, then the heating element was turned on to initiate the temperature ramp. Individuals that were not active after this resting period were excluded from the assay. The rate of temperature increase was 0.3°C per minute at the beginning of the assay, and gradually decreased to approximately 0.1°C per minute (Appendix S1: Figure S2). Individuals were monitored continuously during the ramp until normal movement ceased and the individual no longer responded to gentle stimulus (slight water movement generated by turning the vials). The time at that point was recorded, and the vial was removed from the water bath. The continuous temperature record from the Arduino loggers was used to determine CTmax based on these recorded times. For the copepods examined during this study, the transition from active swimming to immobilization is rapid. With 10 individuals under constant observation, it is unlikely that any individual was at or past its CTmax for more than 1 min—with a ramping rate of approximately 0.1°C per minute at the end of the assays, our estimates of CTmax are unlikely to overestimate this value by more than 0.1°C.

After all individuals had reached their CTmax, the copepods were transferred to the lid of a 96‐well plate and examined using a stereoscope (Olympus SZX7). Individuals were identified to the species level (LaMay et al., 2013; Ward & Whipple, 1959) and categorized as either male, female, or juvenile. For females bearing egg sacs, clutch size (the number of eggs contained) was also recorded. Finally, each individual was photographed along with a scale micrometer for body size measurements. Body size was measured as prosome length using ImageJ (Schindelin et al., 2012). While we did not explicitly monitor survival following the CTmax assays due to logistical constraints on housing individuals after the assays, we note that >95% of the individuals were actively swimming before being photographed, reinforcing that this approach estimates CTmax as opposed to other metrics (such as upper lethal temperatures).

After the entire data set was collected, these images were also re‐analyzed to identify three other potential traits of interest (the presence of developing eggs as a proxy for active energetic investment in reproduction, the presence of lipid droplets as a general proxy for individual condition and energetic reserves, and visually apparent internal parasites or pathogens). There were several diverse pathogen classes observed across the sampling period. Since specific identification was not possible from the images, we grouped these pathogen phenotypes into three visual classes: a “spot” phenotype, a “cloudy” phenotype, and an “other” group that contained the remaining individuals (Appendix S1: Figure S3). Identification of the pathogenic species or physiological processes underlying the infection was not possible with our photo‐based approach, but this analysis provides preliminary evidence of which pathogen classes may impact these copepod populations, which can be followed up with more targeted mechanistic investigations (Eliassen et al., 2024). These additional traits were scored by a single investigator to ensure consistency across the entire set of samples.

### Data analysis

All analyses were conducted with R version 4.1.3 (R Core Team, 2022). We used a linear mixed‐effects model to examine the influence of species, collection temperature, and individual sex on CTmax. All interactions were included in this model. Temperature was centered but not scaled before analysis. Since CTmax assays were often performed over the course of several days after collection, we included the number of days copepods spent in the lab before CTmax was measured as a random effect. The species‐specific acclimation response ratios (ARRs) were estimated as the relationship between CTmax and collection temperature for each species from this initial model by calculating the marginal mean of the linear trend (emmeans package; Lenth, 2025); essentially, the ARR represents the slope of the relationship between collection temperature and CTmax for each species, or the change in CTmax per degree change in collection temperature. Given the limited number of species present in the community, the relationship between a species' mean CTmax and the estimates of ARR obtained here were compared against a collection of estimates from a previous data synthesis on copepod thermal limits to better contextualize the patterns observed (Sasaki & Dam, 2021). For the three diaptomid species (*Skistodiaptomus*, *Leptodiaptomus minutus*, and *L. sicilis*), the residuals from this model were regressed against clutch size to examine potential fitness costs associated with increased thermal limits. It was not possible to examine fecundity for the remaining four species, which release their eggs directly into the water column. A secondary model including data just for females examined the effect on CTmax of species and collection temperature (with their interaction), along with the presence of developing eggs, the presence of lipid droplets, and the presence of potential pathogens.

We also used two approaches to examine the potential environmental drivers of acclimation. First, we examined the correlation between CTmax and a number of predictors (the overall mean, median, minimum, and maximum temperature; the mean daily minimum, maximum, and range; and the temperature range and variance) calculated for periods of time ranging from 1 to 60 days before collection. This analysis is largely speculative, so we identified the top predictor‐duration combination for each species based on the maximum correlation coefficient between the predictor and CTmax values. For two species, *L. minutus* and *L. sicilis*, that appear to acclimate rapidly to changes in temperature (i.e., the temperature measured at the time of collection was the best predictor), we also used a distributed lag nonlinear model to examine potential timescales of this rapid response. Distributed lag nonlinear models can be used to examine the association between factors when there is a delay between exposure and the response of interest (Gasparrini, 2014). For instance, distributed lag nonlinear models can be used to model upper thermal limits as a function of not just the temperatures to which individuals are exposed, but the lag times representing when the individual experienced those temperatures (Redana et al., 2024). We used this approach to examine how the magnitude and timing of fluctuations in temperature over the 200 h prior to collection, based on the USGS continuous temperature record, affected organismal thermal limits.

### Vulnerability hindcast

We used a hindcasting approach to examine whether species‐specific acclimation capacities amplify or reduce variation in how community members are affected by environmental variation. Continuous temperature data were retrieved from the USGS sensor for the time period of October 1, 2015, to January 9, 2025. The full decade of data was used to provide a representative sample of the recent inter‐ and intra‐annual variation to which populations have been exposed. These data were used to generate a hindcast of thermal limits for this community of copepods based on three scenarios: (1) no acclimation, (2) shared acclimation capacity across community members, and (3) species‐specific acclimation capacities. In the “no acclimation” scenario, warming tolerance was calculated based on a fixed thermal limit for each species (mean CTmax value). For the “shared acclimation” capacity, a linear regression of CTmax against species and collection temperature (without interaction) was used to predict thermal limits based on the average daily temperature. This scenario therefore allows for variation in thermal limits but not acclimation capacity across species. The final scenario estimated CTmax for each species using the best predictor‐duration combination as determined by the correlation analysis. The formulas used during these hindcasts are provided in Appendix S1: Table S1. For each of the three scenarios, warming tolerance was calculated as the difference between these predicted thermal limits and the maximum daily water temperature. The average minimum annual warming tolerance (i.e., the maximum vulnerability) was calculated for each of the three scenarios to examine the effect on estimates of vulnerability.

## RESULTS

### Community composition

Our weekly sampling method yielded thermal limit data for all six species of calanoid copepods observed in Lake Champlain: *Skistodiaptomus* sp., *L. minutus*, *L. sicilis*, *Epischura lacustris*, *Senecella calanoides*, and *Limnocalanus macrurus*. We also collected a single individual of *Osphranticum labronectum* in early October 2023; to our knowledge, this is the first observation of this species in Lake Champlain. There is also some uncertainty regarding the *Skistodiaptomus* species we collected. In Lake Champlain, this has historically been identified as *Skistodiaptomus oregonensis* (LaMay et al., 2013), but a small number of individuals we collected in summer 2024 were identified as *S. pallidus* via genetic barcoding (cytochrome oxidase I) as part of a separate project. *S. pallidus* does occur in nearby water bodies (Chiapella et al., 2021) and has previously been noted for its potential to invade Lake Champlain from adjacent water bodies (Malchoff et al., 2005). As we did not preserve material from our CTmax assays and it is not possible to differentiate these two species in the images taken, we will refer to *Skistodiaptomus* sp. throughout this study. We acknowledge that the data reported are likely for a combination of the two species. Finally, for one of the winter species, *L. sicilis*, we detected the presence of two distinct size morphs (Appendix S1: Figure S4). Dissection of several adult individuals from both morphs confirmed a shared species identity (Ward & Whipple, 1959); instead, the presence of these distinct size classes likely indicates the transition between different generations in the population (Herzig et al., 1980; Lai, 1977).

### Seasonality, phenology, and thermal limits

Surface temperatures measured at the time of collection ranged from 2.5 to 26.5°C during the sampling period, which included 48 unique collection events (Figure 1). We measured a total of 1312 individual thermal limits during this period. All species (except *O. labronectum*) were collected multiple times, ranging from 13 to 40 collections (*S. calanoides* and *L. minutus*, respectively; Table 1). For all species, thermal limit variation across individuals within a collection tended to be relatively small (SD typically <2°C, maximum value of 4.1°C; Appendix S1: Figure S5). While our sampling approach precludes quantitative estimates of abundance, the number of CTmax measurements made for each species does qualitatively reflect their relative abundance in the samples collected.

**FIGURE 1:**
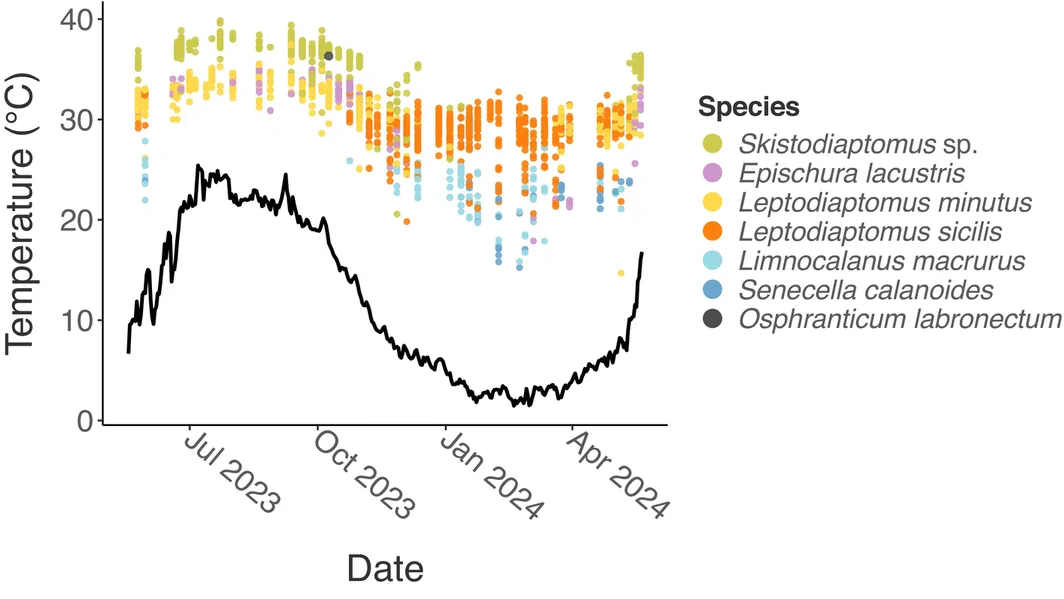
Seasonality in critical thermal maximum (CTmax) and lake surface temperature. Individual CTmax measurements are shown as points, with the different species shown in different colors. Lake surface temperature from a United States Geological Survey continuous temperature sensor is shown with the solid line.

**TABLE 1:** Details about sample sizes for each of the seven species examined in this study.

Species	Overall *N*	Collections	Average *N*
*Leptodiaptomus sicilis*	482	29	16.62
*Leptodiaptomus minutus*	324	40	8.10
*Skistodiaptomus* sp.	275	31	8.87
*Epischura lacustris*	102	26	3.92
*Limnocalanus macrurus*	86	16	5.38
*Senecella calanoides*	42	13	3.23
*Osphranticum labronectum*	1	1	1

*Note*: Overall *N* shows the total number of individuals across all collections. The number of collections with the species present and the average number of CTmax measurements per collection for each species are also included.

The calanoid copepod community could be divided into a Summer–Fall group (*E. lacustris*, *Skistodiaptomus* sp., and *L. minutus*) and a Winter–Spring group (*S. calanoides*, *L. macrurus*, and *L. sicilis*) (Figure 2A). The Summer–Fall group is characterized by smaller body size and higher thermal limits, while the Winter–Spring group included species with both small and large body sizes, but lower thermal limits (Appendix S1: Figure S6). *O. labronectum* had a relatively high upper thermal limit (36.3°C), and groups with the Summer–Fall assemblage. With only a single observation, however, this affinity is tentative. Across all species, CTmax increased with increasing collection temperatures (discussed more in the following section; Figure 2B). A notable exception to this trend is the increase in *L. sicilis* CTmax during mid‐winter, associated with the transition between the two generations. This is potentially related to differences in developmental temperature between the different generations. Warming tolerance (the difference between CTmax and collection temperature) decreased with increasing collection temperatures for all species, suggesting increasing vulnerability at higher temperatures in both assemblages (Figure 3A).

**FIGURE 2:**
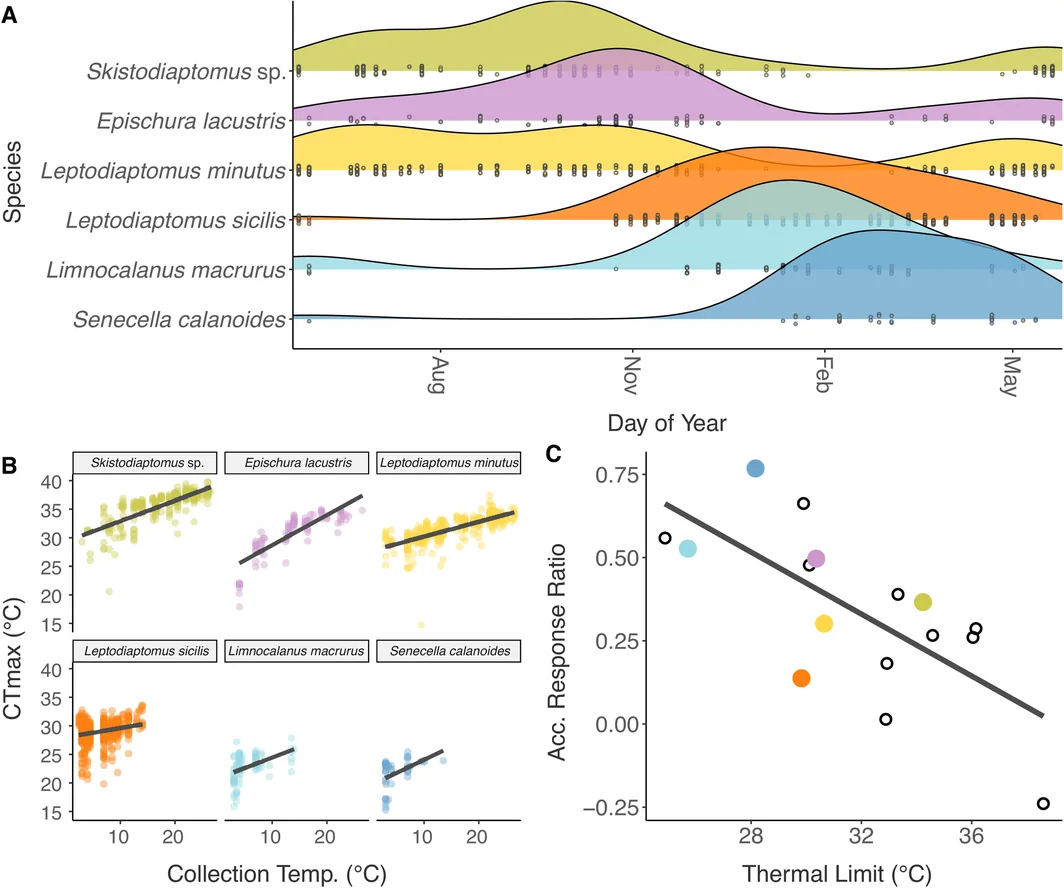
(A) Ridge plots showing the seasonal occurrence of the six major species of calanoid copepods in Lake Champlain. These plots show the temporal distribution of critical thermal maximum (CTmax) measurements for each species, with the height of each plot indicating the relative frequency of measurements within each species throughout the year. (B) The relationship between CTmax and the surface temperature recorded at the time of collection. CTmax values for each species are shown separately, along with a linear regression describing the relationship between measured thermal limits and collection temperature. (C) The negative relationship between acclimation response ratios and upper thermal limits. Values from Lake Champlain species measured in this study are shown in color, while values from a previous data synthesis study (Sasaki & Dam, 2021) are shown as empty circles.

**FIGURE 3:**
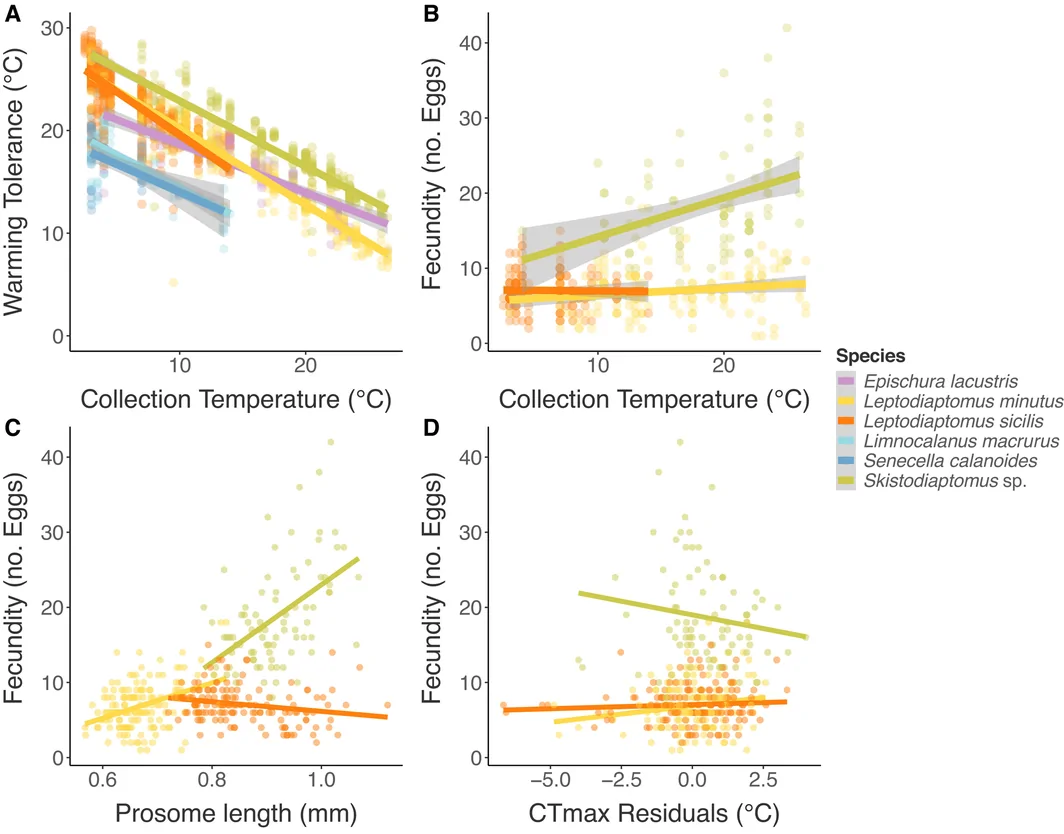
Observed values for warming tolerance (A) and egg clutch size (B) plotted against collection temperature, along with the relationships between fecundity and collection temperature (C) and thermal limit residuals (D). Each point represents an individual measurement, with different species shown in different colors. A regression line is included for each species. Critical thermal maximum (CTmax) residuals show variation in CTmax after controlling for the effect of collection temperature. Fecundity data are shown for only the three egg‐sac‐bearing species in the data set.

Clutch size was not related to collection temperature in the two *Leptodiaptomus* species, but increased with collection temperature in the *Skistodiaptomus* sp. (Figure 3B). Clutch sizes tended to increase with female body size in *Skistodiaptomus* sp. and *L. minutus*, but not in *L. sicilis* (Figure 3C). There were no strong relationships between CTmax residuals and clutch size in any species, indicating that increases in thermal limits were not influenced by a trade‐off with reproductive output (Figure 3D).

The second model examined the effect of developing eggs, lipid reserves, and visible pathogens on female CTmax. Model selection indicated no change in model fit when the presence of developing eggs and the presence of lipid droplets were removed as factors. Analysis of just the model including species, collection temperature, their interaction, and the presence of visible pathogens indicated a significant effect of pathogen presence on female CTmax. Estimated marginal means for the three different pathogen classes indicate variability in the effect on CTmax relative to individuals without visible pathogens (Appendix S1: Figure S7). There was no change in CTmax in individuals exhibiting the “spot” phenotype, whereas copepods exhibiting phenotypes in the other two classes exhibited a decrease in CTmax of approximately 2°C, although the effect of the “other” pathogen class had a large estimated SE.

### ARRs, drivers, and timescales

CTmax increased with increasing collection temperatures for all species, suggesting widespread effects of acclimation (Figure 2B). We also observed similar changes in CTmax regardless of whether the lake was warming or cooling (i.e., symmetrical changes in CTmax during the spring–summer transition and the summer–fall transition; Appendix S1: Figure S8). This reinforces that these changes are driven by acclimation rather than processes like rapid adaptation or environmental filtering of standing genetic variation, which would likely have led to an asymmetry in the response to warming and cooling. If the changes in CTmax were driven by selection for warm‐tolerant genotypes as lake temperatures increased, for example, we would expect this loss of genetic diversity to flatten the relationship between CTmax and collection temperature during the fall as water temperatures decrease.

While the relationship between CTmax and collection temperature was positive for all species, the observed acclimation effects varied in magnitude across species. Species‐specific ARR estimates, or the change in CTmax per degree change in collection temperature, varied from 0.14 to 0.77 (*L. sicilis* and *S. calanoides*, respectively). The magnitude of the ARR estimates tended to decrease with increasing average thermal limits (Figure 2C), mirroring the pattern observed across other copepod taxa (Sasaki & Dam, 2021), suggesting a potential trade‐off between acclimation capacity and baseline thermal tolerance.

The ARR estimates are based on the relationship between collection temperature and CTmax. We also examined more specific potential drivers of acclimation responses (Figure 4). For three of the species (*E. lacustris*, *S. calanoides*, and *Skistodiaptomus* sp.), CTmax was most strongly correlated with the maximum temperature. In contrast, for *L. macrurus*, CTmax was most strongly correlated with minimum temperature, while the two *Leptodiaptomus* species had CTmax values that were most strongly correlated with collection temperature. There was also substantial variation in the duration of time that best explained CTmax variation, ranging from an almost 30‐day period in *E. lacustris* to <1 day in the two *Leptodiaptomus* species. We note, however, that for all species except *S. calanoides*, the difference in correlation coefficients between the strongest predictor and collection temperature was negligible (Figure 4B).

**FIGURE 4:**
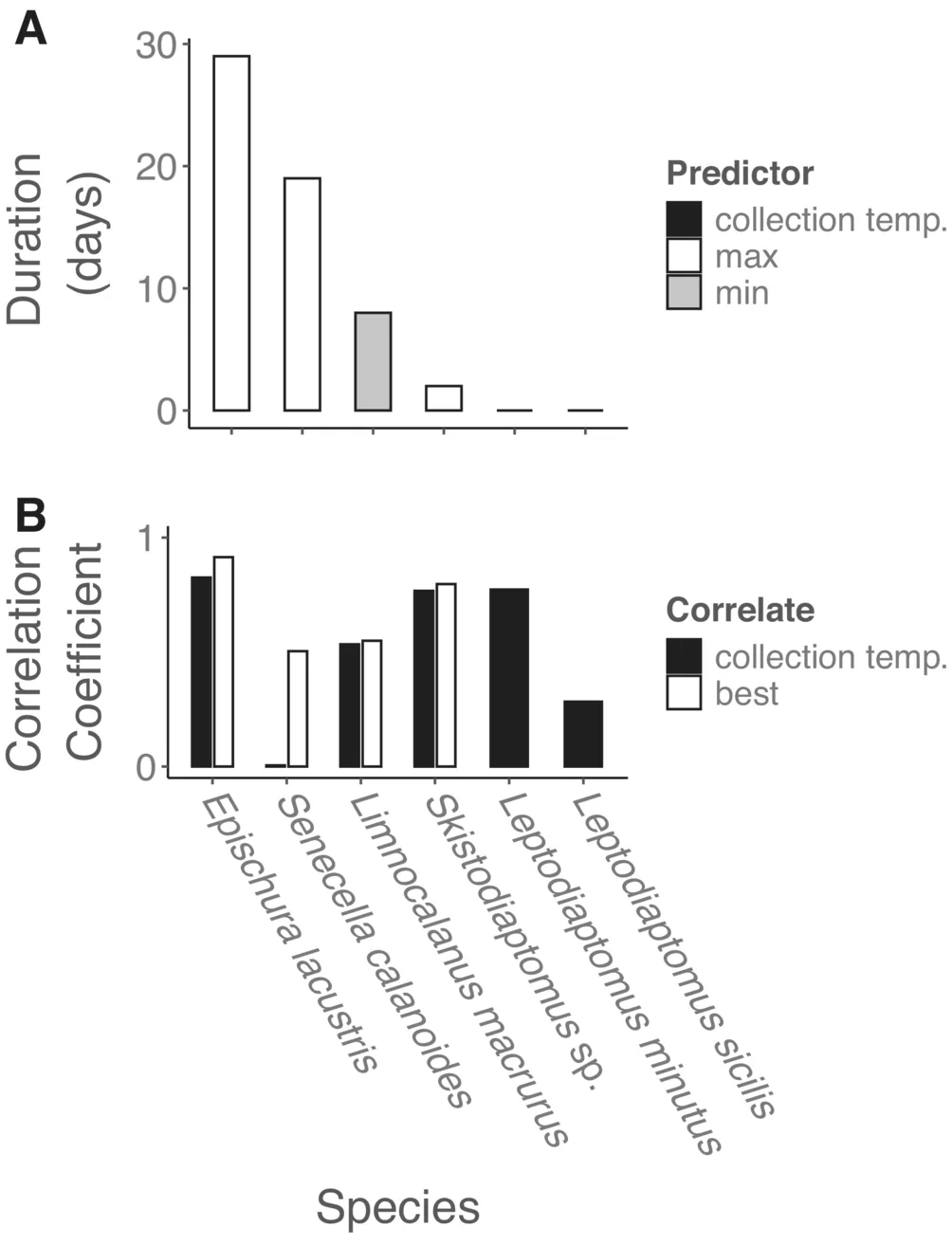
(A) The duration of potential acclimation periods (days prior to collection) shown for each of the species. Bar fill color indicates the potential cue (collection temperature, maximum temperature during the acclimation period, or minimum temperature during the acclimation period). (B) The observed correlation coefficients between the best predictor (white bars) and the temperature measured at the time of collection (black bars). Note that the order of species is the same across both panels.

The two *Leptodiaptomus* species present in Lake Champlain both appear to adjust CTmax rapidly, with CTmax most closely correlated with temperature at the time of collection. We used a distributed lag nonlinear model to examine the potential timescales of this rapid acclimation. For both species, these models indicated acclimation to short‐term deviations (spikes or drops in temperature) occurring during the 200 h leading up to collection, with the change in CTmax depending on both the magnitude of the deviation and the timing (Appendix S1: Figure S9). Large deviations (+4 or −4°C) during the 2 days leading up to collection increased or decreased CTmax by approximately 0.5°C, respectively. The effect of these large deviations diminished as timing prior to collection increased. The effect also decreased as the magnitude of deviation decreased—changes of ±1°C had only minor effects on CTmax even when the deviation occurred near the time of collection.

### Vulnerability hindcast

The three acclimation scenarios (no acclimation, shared acclimation, species‐specific acclimation) yielded different estimates of vulnerability across the community (Figure 5; Appendix S1: Figure S10). In all cases, warming tolerance was lowest in the “no acclimation” scenario. Acclimation increased warming tolerance in all species, but there was variation in how species‐specific acclimation capacity affected vulnerability. While species‐specific acclimation capacities increased warming tolerance (reduced estimated vulnerability) in some species, the effect was minimal or negative in others. Within the winter‐dominant species, for example, *Leptodiaptomus sicilis* had a reduced warming tolerance when species‐specific acclimation capacity was included, while *S. calanoides* and *L. macrurus* both had a slightly increased warming tolerance.

**FIGURE 5:**
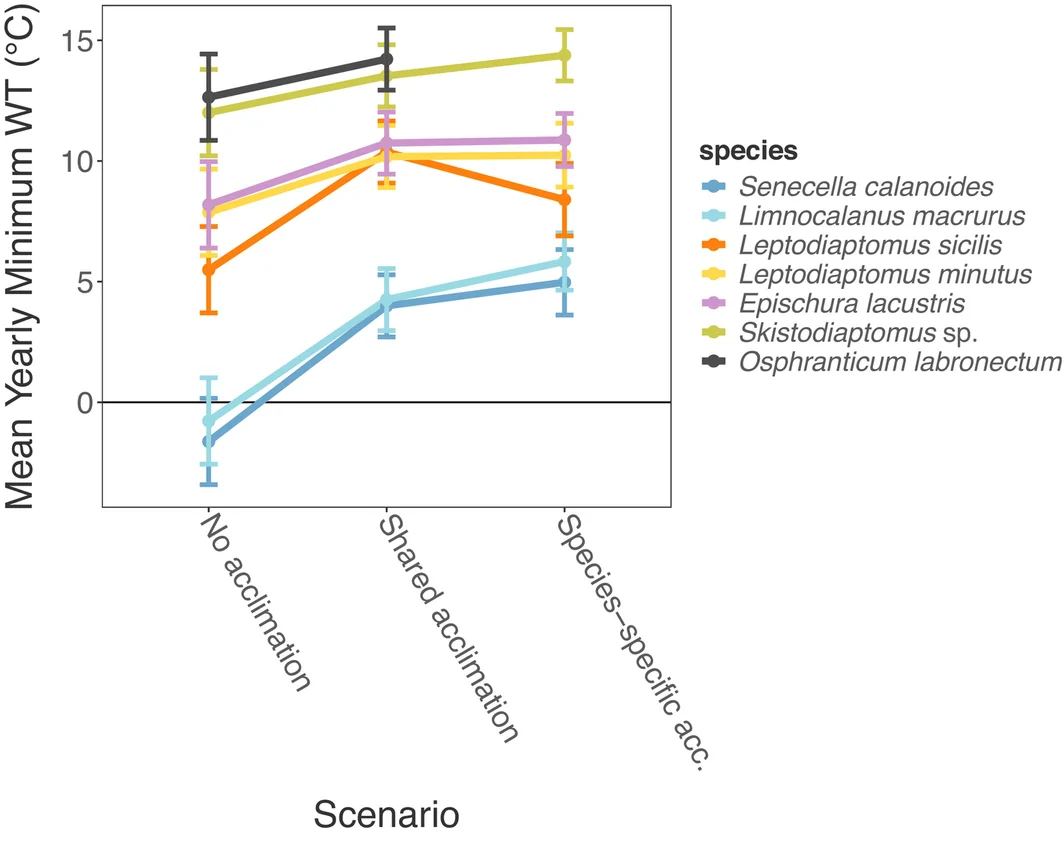
Average annual minimum warming tolerance estimated for each species in the community based on three thermal limit hindcast scenarios. The “no acclimation” scenario assumes fixed thermal limits for each species. A shared acclimation capacity scenario assumes thermal limits vary across species as well as in response to average daily temperature, with a shared acclimation capacity across all community members. The species‐specific acclimation scenario allows both thermal limits and acclimation capacity to vary across species based on the predictor‐duration analysis described in Figure 4.

## DISCUSSION

We examined seasonal variation in the upper thermal limits of the community of calanoid copepods in Lake Champlain. By sampling across the entire annual temperature range, we revealed substantial variation in thermal limits between the seasonal community assemblages as well as within species, providing estimates of acclimation capacity under natural conditions. Our results show that acclimation has a significant but variable effect on thermal limits across the entire community, and reinforce that temporal variation in thermal limits can mediate population vulnerability to warming. At the community level, variation in both thermal limits and acclimation capacity are important factors to consider when assessing ecological vulnerability.

There are clear differences in thermal limits between the seasonal assemblages of calanoid copepods in Lake Champlain. As expected, summer‐dominant species had substantially higher thermal limits than those species most abundant during the winter. While these differences between assemblages may be driven by changes in temperature across seasons, measurements on field‐acclimated individuals may also reflect the influence of a range of environmental factors. Copepod thermal limits are, for example, reduced under food limitation (Rueda Moreno & Sasaki, 2023). Since food abundance (and possibly quality) is likely lowest when water temperatures are warmest (due to increased stratification, diminishing nutrient concentrations in the surface layer, increase in toxic/non‐palatable species like Cyanobacteria, etc.; Mihuc et al., 2024), if food‐driven effects alone explained variation in CTmax, we would expect the trend to be reversed, with CTmax decreasing with increasing temperatures. Copepods are also highly sensitive to photoperiod (Hairston & Dillon, 1990). In natural settings, temperature and photoperiod can be highly correlated, and as such we are unable to disentangle the potential effects of photoperiod as a potential cue for thermal acclimation (Mahoney & Hutchison, 1969). Laboratory experiments explicitly examining the potentially interacting effects of factors like temperature, food abundance/quality, and photoperiod would be appropriate areas for future research.

Regardless of cause, differences in thermal limits may be informative about long‐term trends in competitive interactions (Martin & Ghalambor, 2023) and abundance under warming (Roeder et al., 2021). While there have been large changes in the abundance of calanoid copepods in Lake Champlain over the past decades (Mihuc et al., 2012), the patterns we observed here suggest that the copepod populations are not directly limited by temperature, with warming tolerance (the difference between thermal limits and environmental temperatures) remaining above 10°C, even during the transition between assemblages. Seasonal diapause or dormancy may be initiated well before the onset of stressful temperatures, thus buffering species against temperature‐induced mortality over both intra‐ and inter‐annual timescales. Alternatively, seasonal occurrence and long‐term changes may be more strongly shaped by changes in temperature relative to species' thermal optima, the temperature where organismal performance is maximized. While the experiments required to determine thermal optima typically have larger time and equipment requirements, CTmax is often strongly correlated with thermal optimum (Cicchino et al., 2026) and is relatively easy to scale across seasons and species. As such, CTmax is a useful metric for at least identifying scenarios where thermal performance may shape population dynamics. Another interesting potential future direction would be integration of the CTmax data collected here with the thermal tolerance landscape approach (Rezende et al., 2014). With additional experimental work to estimate the required scaling parameters, this could examine how both exposure magnitude and duration affect survival in these populations (Rezende et al., 2020). Finally, over long timescales, population responses will also be affected by evolutionary responses—the data collected here provide a baseline for similar surveys over the coming years, which may provide key insights into population responses to climate change (Ardelan et al., 2023; Buckley et al., 2025).

In addition to variation in thermal limits, there was notable variation in acclimation capacity across species. While the observation of strong acclimation responses under natural conditions highlights the potential importance of this mechanism in shaping responses to climate change, robust predictions about the role of acclimation as conditions change will depend on identifying the environmental driver of acclimation responses (Dowd & Denny, 2020). Our results suggest that different cues over different time spans drive the patterns in thermal acclimation we observed. Across the species we examined, thermal limits correlated with different aspects of the thermal environment (e.g., the minimum or maximum temperatures) over timescales from <1 to 30 days. For the two *Leptodiaptomus* species, which appear to acclimate most rapidly, distributed lag nonlinear models suggest responses to temperature variation on the order of hours. These drivers and time spans provide hypotheses that can be tested in laboratory acclimation experiments (Kraskura et al., 2024), but already point to the importance of understanding the species‐specific drivers of acclimation to accurately predict the role of plasticity in mediating vulnerability over short and long timescales. Indeed, the hindcasted warming tolerance values suggested species‐specific acclimation dynamics can alter estimated vulnerability across this community. Interestingly, our results also indicate that aspects of temperature variability (range, variance, etc.) are not the primary drivers of acclimation in these copepods. Similar results were obtained for another freshwater calanoid in a set of mesocosm experiments (Bonadonna et al., 2025). These manipulative experiments balance experimental feasibility and environmental realism (Sasaki et al., 2025), and may play a large role in testing both species‐ and community‐level hypotheses about the interactions between environmental variation and plasticity.

The strong effects of temperature acclimation under natural conditions we have shown here also highlight the key role plasticity will play in shaping not only species but community responses to warming. Two commonly predicted community responses to warming are phenological shifts and thermophilization (replacement of cold‐adapted species by warm‐adapted species) (Inouye, 2022; McLean et al., 2021). Both predictions are rooted in the assumption that species tend to exhibit niche conservatism, or that seasonal and geographic occurrences will shift to maintain species within a consistent thermal niche (Wiens et al., 2010). Rapid acclimation to changes in temperature violates this assumption, or at the very least requires a more dynamic perspective on the processes underlying potential niche conservatism, particularly as large‐scale examinations suggest intraspecific niche divergence may be common in planktonic copepods (McGinty et al., 2021). Populations with strong acclimation capacity may not respond to warming by shifting seasonal or geographic occurrence, relying instead on acclimation to rapidly shift the bounds of the fundamental or realized niche. From this perspective, the importance of examining population and species variation in acclimation responses is clear—if acclimation capacity mediates these dynamics at the population level, variation in acclimation capacity across community members may result in inconsistent patterns in changes in abundance, with the magnitude of spatial or temporal shifts of the individual community members inversely correlated with the magnitude of their ability to acclimate to new conditions. In accordance with this, strong acclimation capacity may influence the observed complexity in phenological shifts in copepod communities over the past several decades (Corona et al., 2024). This might also contribute to surprising observations of “borealization” (increasing presence of cold‐adapted species) in zooplankton communities (Khaliq et al., 2024), if acclimation capacity increases with decreasing thermal limits (as observed here as well as across copepod taxa more generally; Sasaki & Dam, 2021). Studies examining acclimation capacity for species assemblages across environmental gradients are needed in order to better understand the interactions between climate change, plasticity, and community dynamics, and in particular are needed to better understand the relative contributions of short‐ and long‐term processes in “ecological acclimation” to climatic changes (Lovell et al., 2023; Stemkovski et al., 2025).

Finally, we note that the fairly intensive sampling regime we employed had clear benefits, capturing data for all species of calanoid copepods previously reported in Lake Champlain, with additional observations of potentially newly introduced species. One of these novel species, *O. labronectum*, is widely distributed throughout freshwater systems across North America, with a range extending from Central America into Canada (Gutierrez‐Aguirre & Suárez‐Morales, 1999). This broad distribution suggests this species is able to tolerate a wide range of conditions, although the role of local adaptation is unknown. The data point we have collected (for whatever a single measurement is worth) suggests a relatively high upper thermal limit, with a higher CTmax value than all but one other species in Lake Champlain. In addition to the observation of *O. labronectum*, we also found evidence for the presence of *S. pallidus* in Lake Champlain. *S. pallidus* is comprised of multiple divergent mitochondrial lineages (Thum & Derry, 2008). The lineage we observed is widespread in the Eastern United States and is known to be invasive in freshwater systems in New Zealand, Mexico, and Germany (Branford et al., 2017). What proportion (if any) of the *Skistodiaptomus* individuals we examined belong to *S. pallidus* as opposed to the historically dominant *S. oregonensis* we are unable to determine. A targeted study combining COI barcoding with individual thermal limits could disentangle any species‐specific patterns in CTmax and examine the realized seasonal/thermal niche of *S. oregonensis* and *S. pallidus* in Lake Champlain, providing insights into invasion dynamics with congeneric species. It is worth noting that both novel observations (*O. labronectum* and *S. pallidus*) have the highest thermal limits of the calanoid species in this community—high upper thermal limits may play a role in the success of invasive or newly introduced species (Kelley, 2014). It would be interesting to compare upper thermal limits for notable invasive calanoid copepod species in other regions (e.g., *Arctodiaptomus dorsalis*, *Pseudodiaptomus marinus* and *P. forbesi*, and *Sinodiaptomus sarsi*; Orsi & Walter,^1991^; Reid, 2007; Svetlichny & Samchyshyna, 2021; Uttieri et al., 2020) with those of the copepod communities in their native and invaded ranges to test whether high thermal limits are predictive of invasion potential.

## CONCLUSIONS

The effects of climate change on the distribution of biodiversity across the planet are a major concern. Here, we show that acclimation has important effects on organismal thermal limits under natural conditions and can drive seasonal changes that mediate vulnerability to warming over both short and long timescales. Only a handful of studies, however, have examined community‐wide variation in acclimation responses over the full annual temperature range. Our results show strong divergence of thermal limits in line with expectations about seasonal occurrence—thermal limits are lower in winter‐dominant species than in summer‐dominant species. Most importantly, we show that acclimation produces substantial intraspecific variation under natural conditions that can play a key role in shaping community responses to climate change. Seasonal acclimation links individual‐, population‐, and community‐level processes, and needs to be incorporated into predictions about responses to climate change.

## CONFLICT OF INTEREST STATEMENT

The authors declare no conflicts of interest.

## Supporting information


Appendix S1.

## Data Availability

Data and code (Sasaki, 2026) are available in Zenodo at https://doi.org/10.5281/zenodo.22097490.
