# Supplementary material for: A high‐resolution physiological time‐series uncovers strong but variable seasonal acclimation of copepod thermal limits
Source: Ecology. 2026 Oct 26;107(10):e70564. doi: 10.1002/ecy.70564 (PMC13696977; doi:10.1002/ecy.70564)
Supplement: Supplementary file 1 [file ECY-107-e70564-s001.pdf]

Appendix S1

A high-resolution physiological time-series uncovers strong but variable seasonal acclimation of copepod thermal limits

Matthew Sasaki, Allison Rodger, Chanchal Saratkar, Jamie Cull-Host, Sydney Sharp, Melissa Pespeni

Ecology

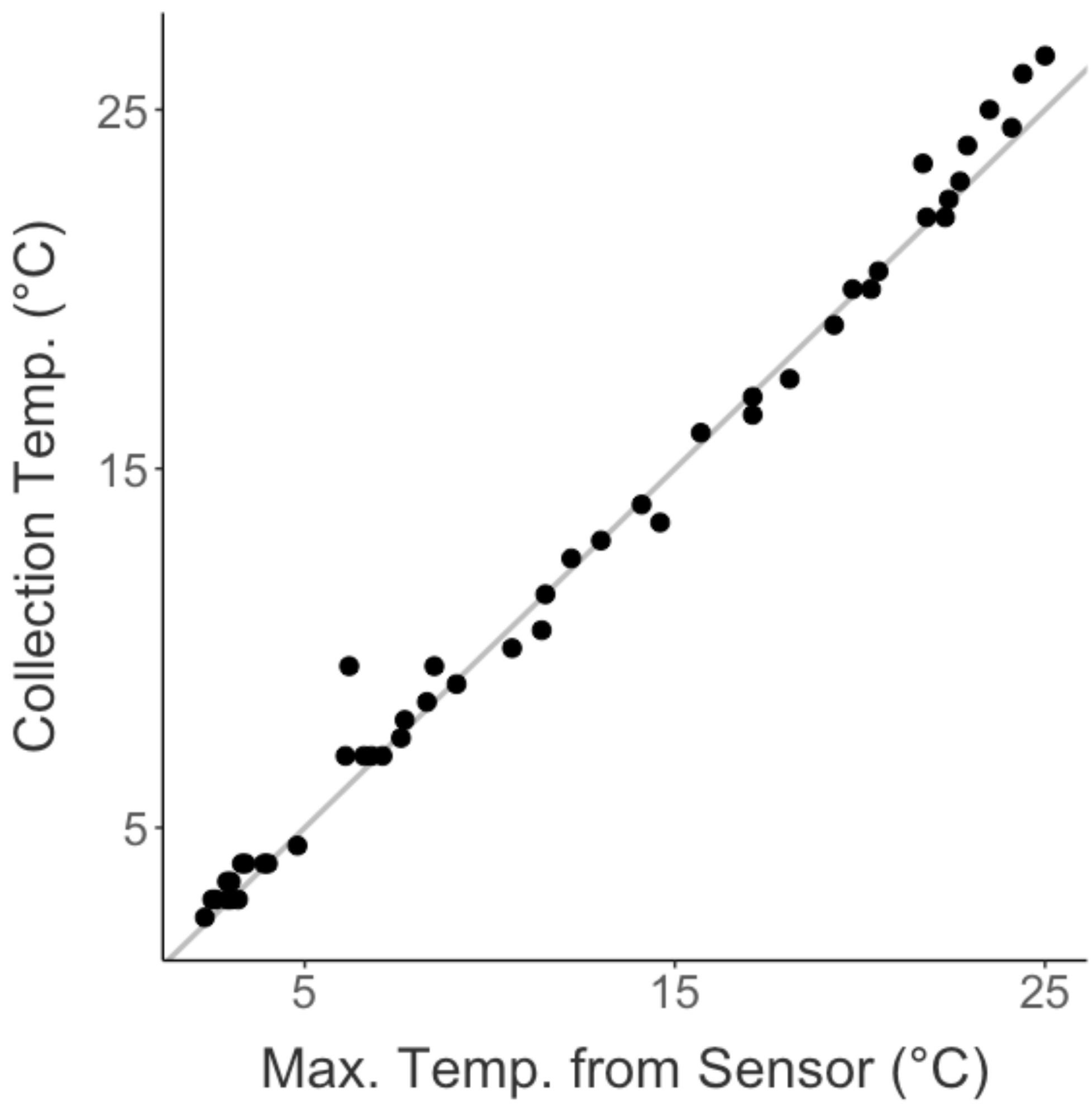

Figure S1 – Correlation between the temperature measured at the time of collection and the maximum daily temperature recorded by the nearby USGS continuous temperature sensor.

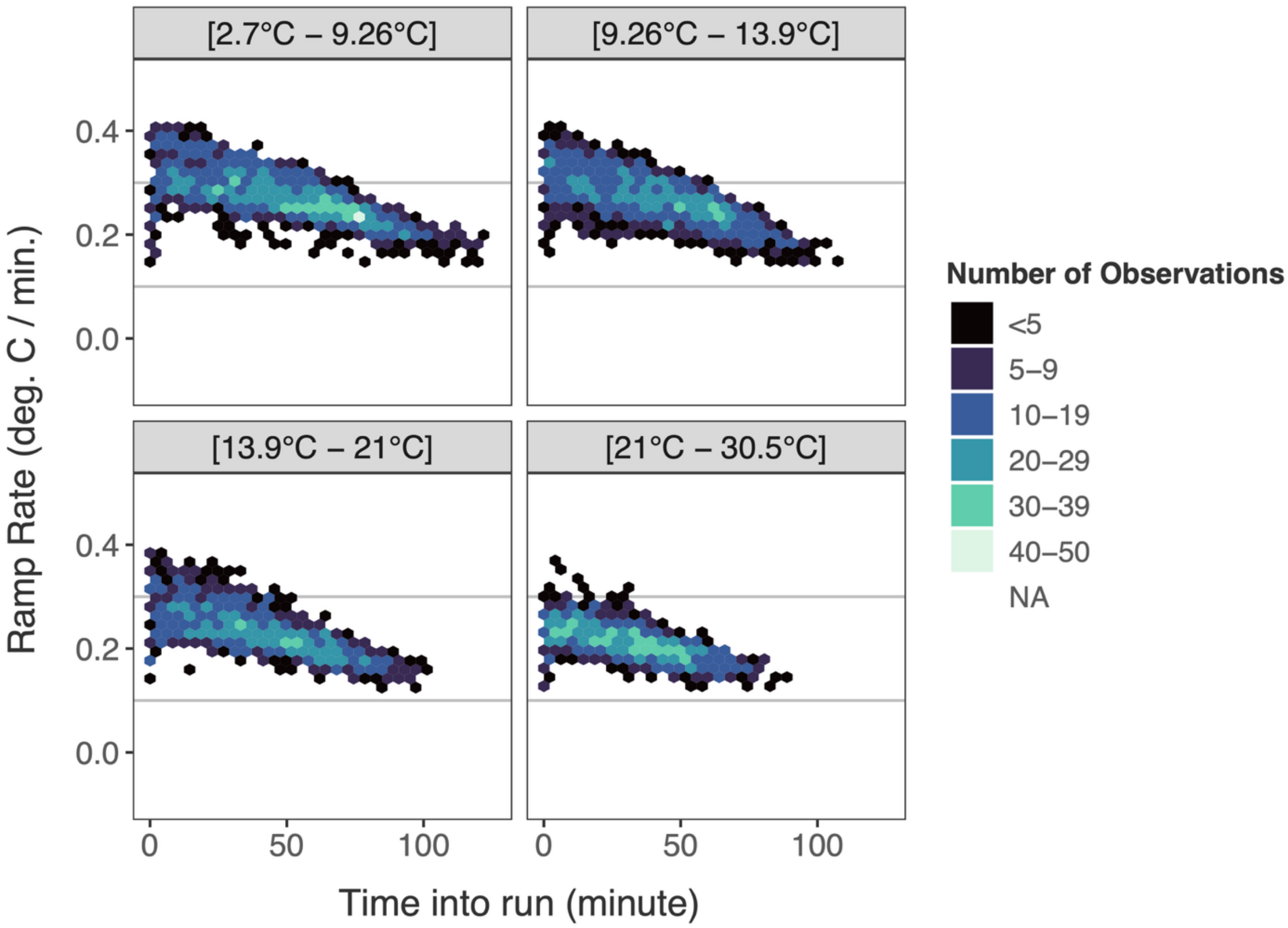

Figure S2 – A summary of the temperature ramping rates observed over the course of the experiments. Ramping rates (change in water temperature per minute) were calculated over minute intervals for each assay. Individual assays were binned into four groups based on their starting temperatures. Ramping rates are plotted over time, with shading representing the number of observations. Bins with a single observation are omitted.

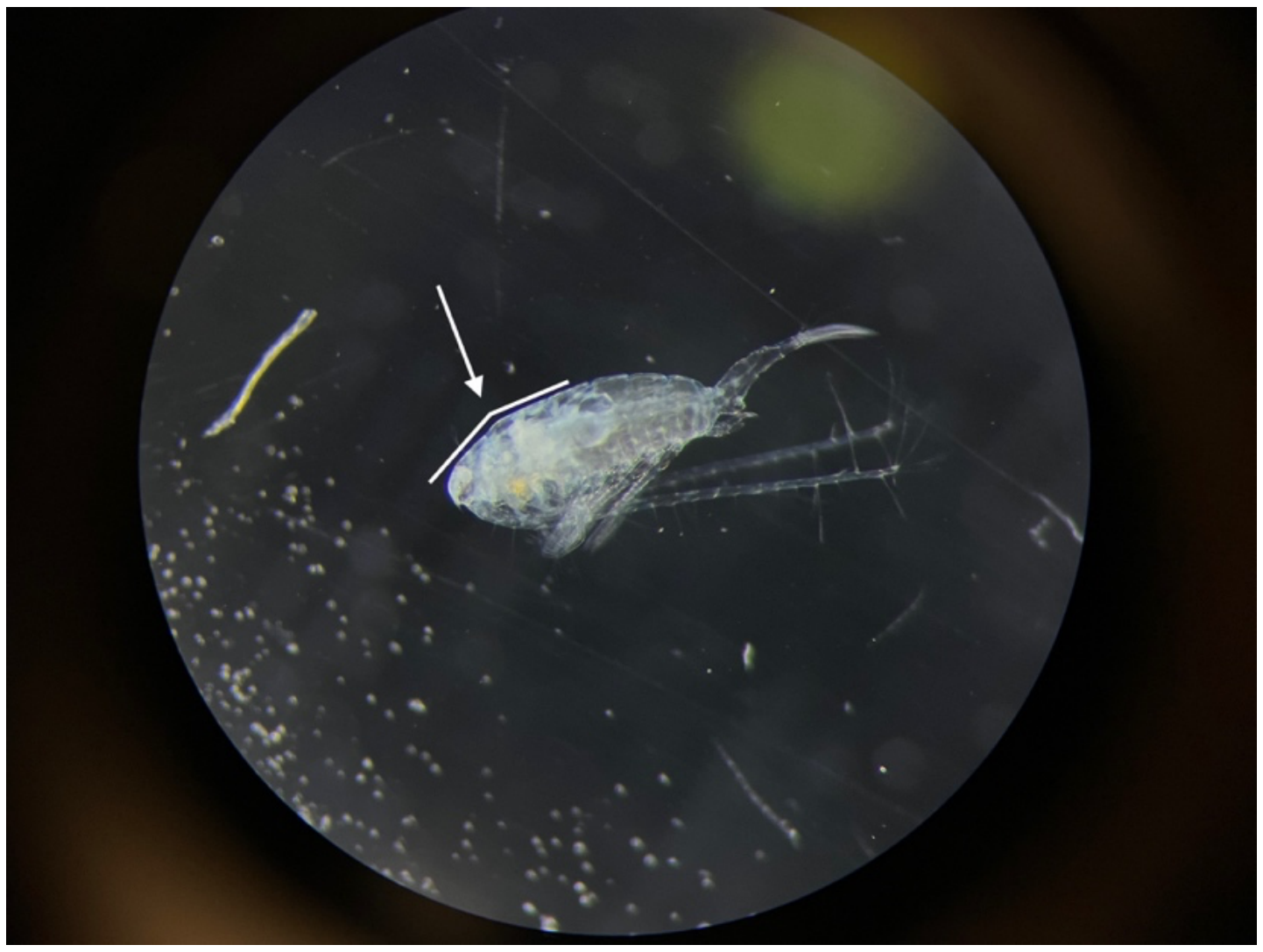

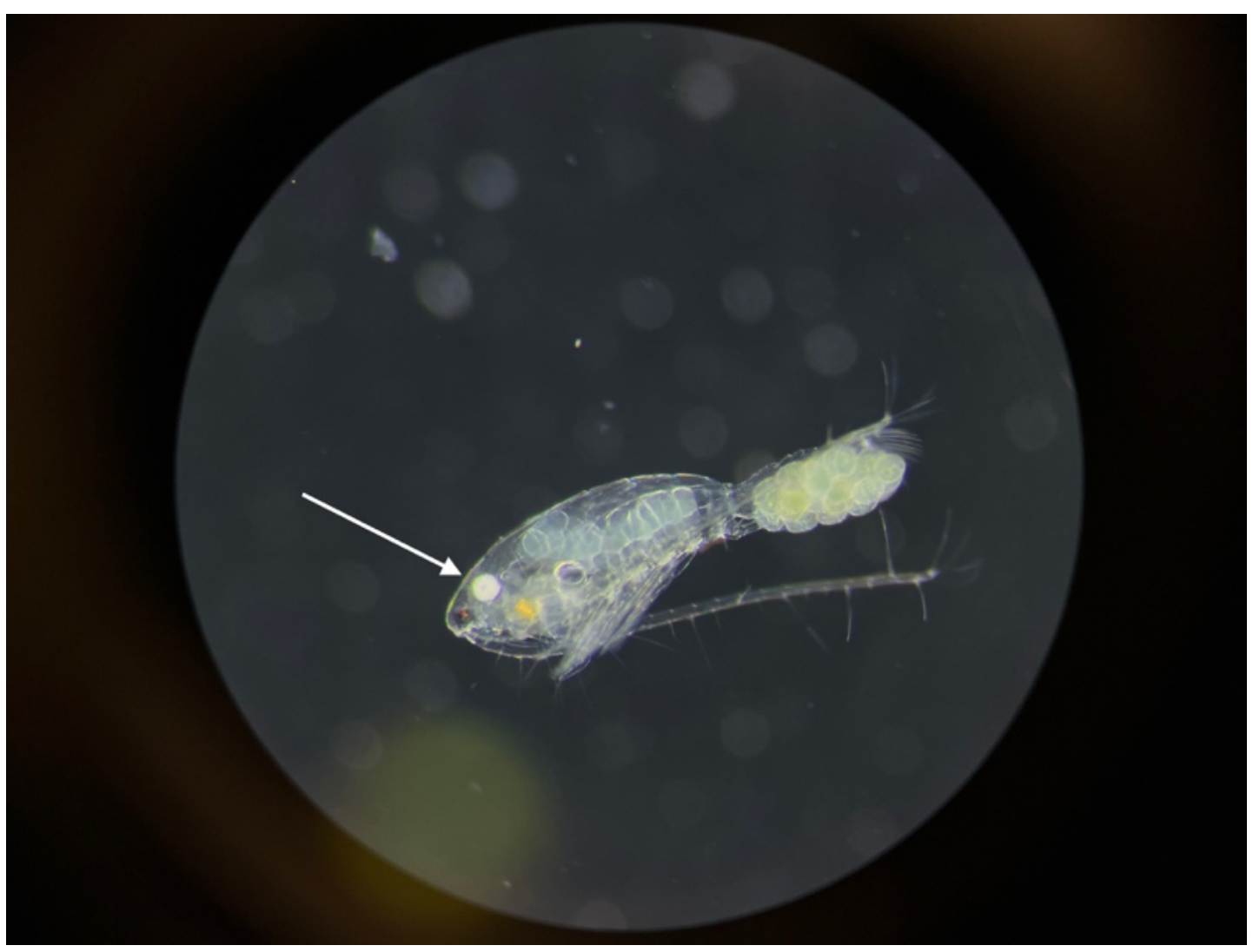

Figure S3 – Images of copepods with the 'spot' and 'cloudy' pathogen phenotypes. All other phenotypes are grouped together in 'other'. Arrows added to highlight the relevant phenotypic features. Image credit: Matthew Sasaki.

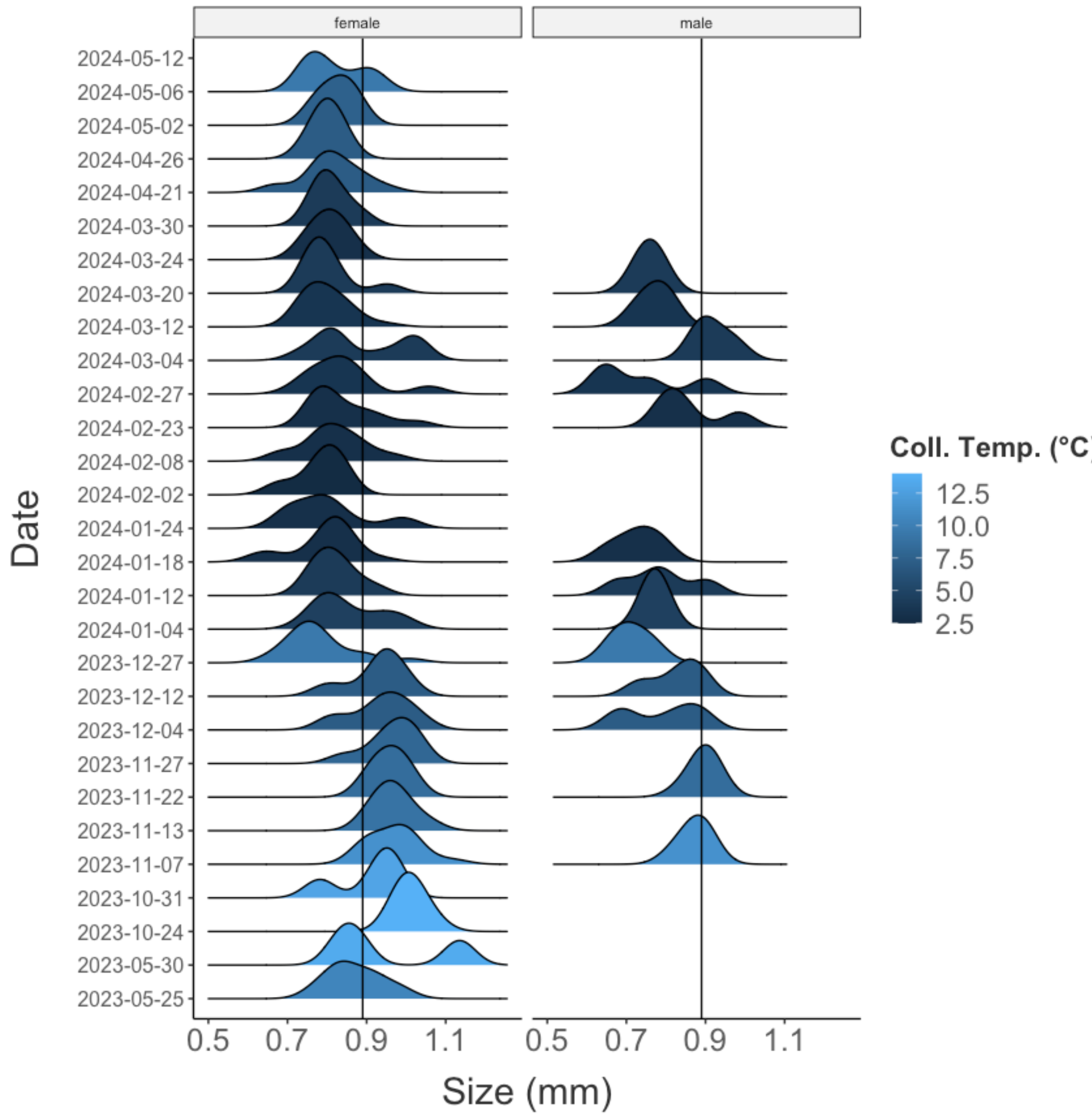

Figure S4 – Leptodiaptomus sicilis size distributions for each sample date, highlighting the presence of the two distinct size morphs throughout the sampling period.

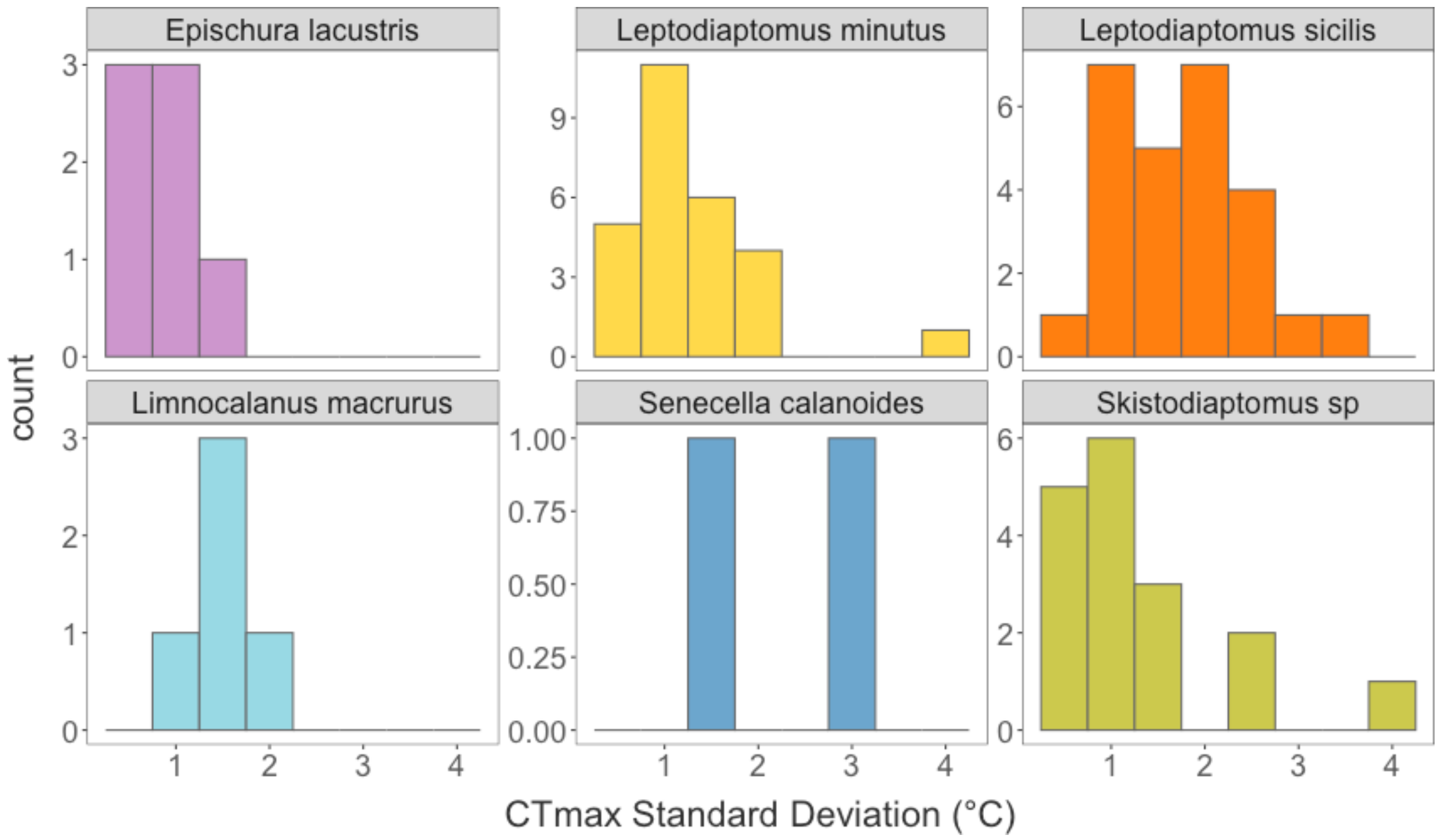

Figure S5 – The distribution of CTmax standard deviations for the different collections of each species.

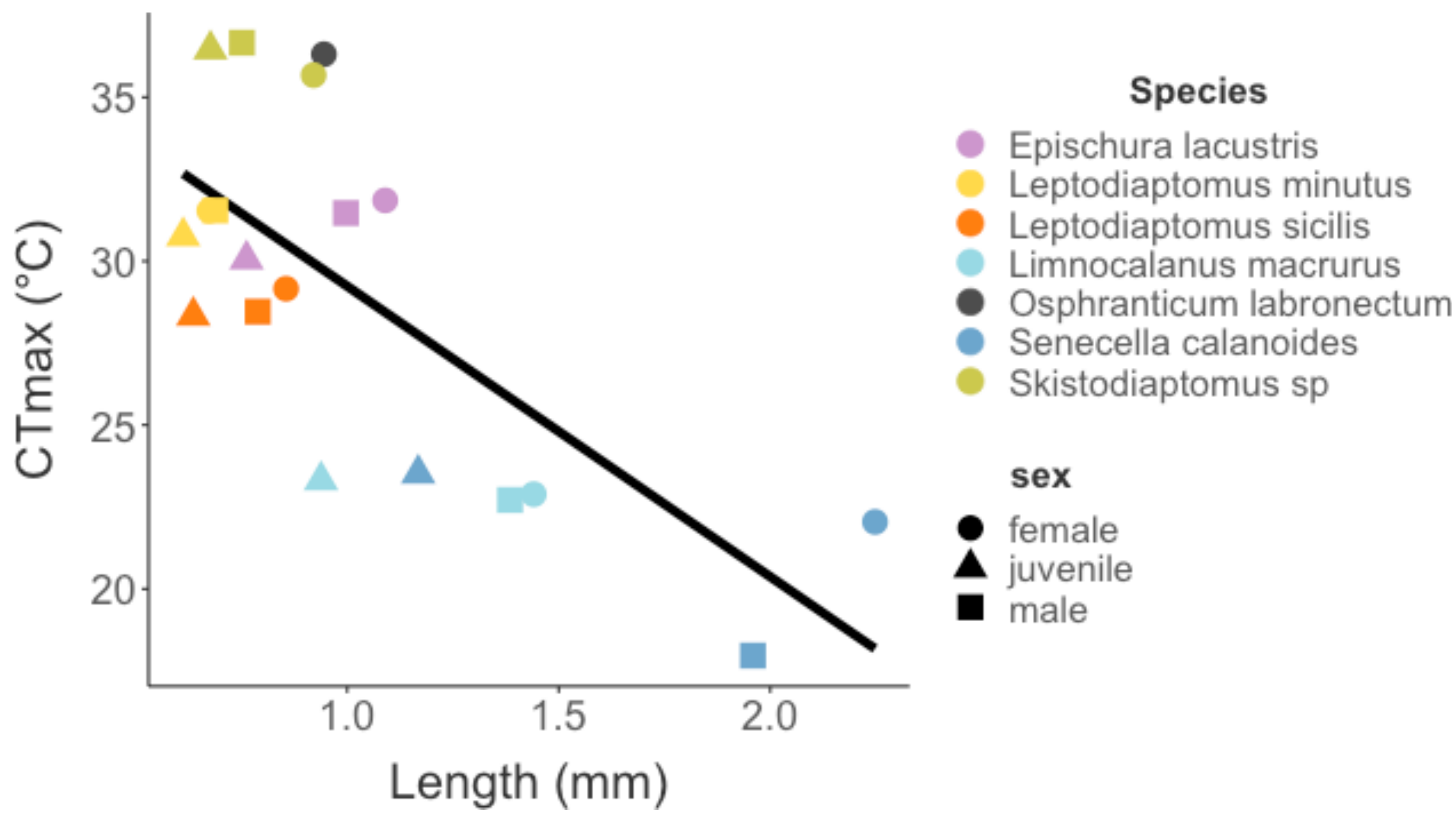

Figure S6 – CTmax tends to decrease with increasing size. Mean values for both CTmax and size (measured as prosome length) are shown here for male, female, and juvenile individuals from each species.

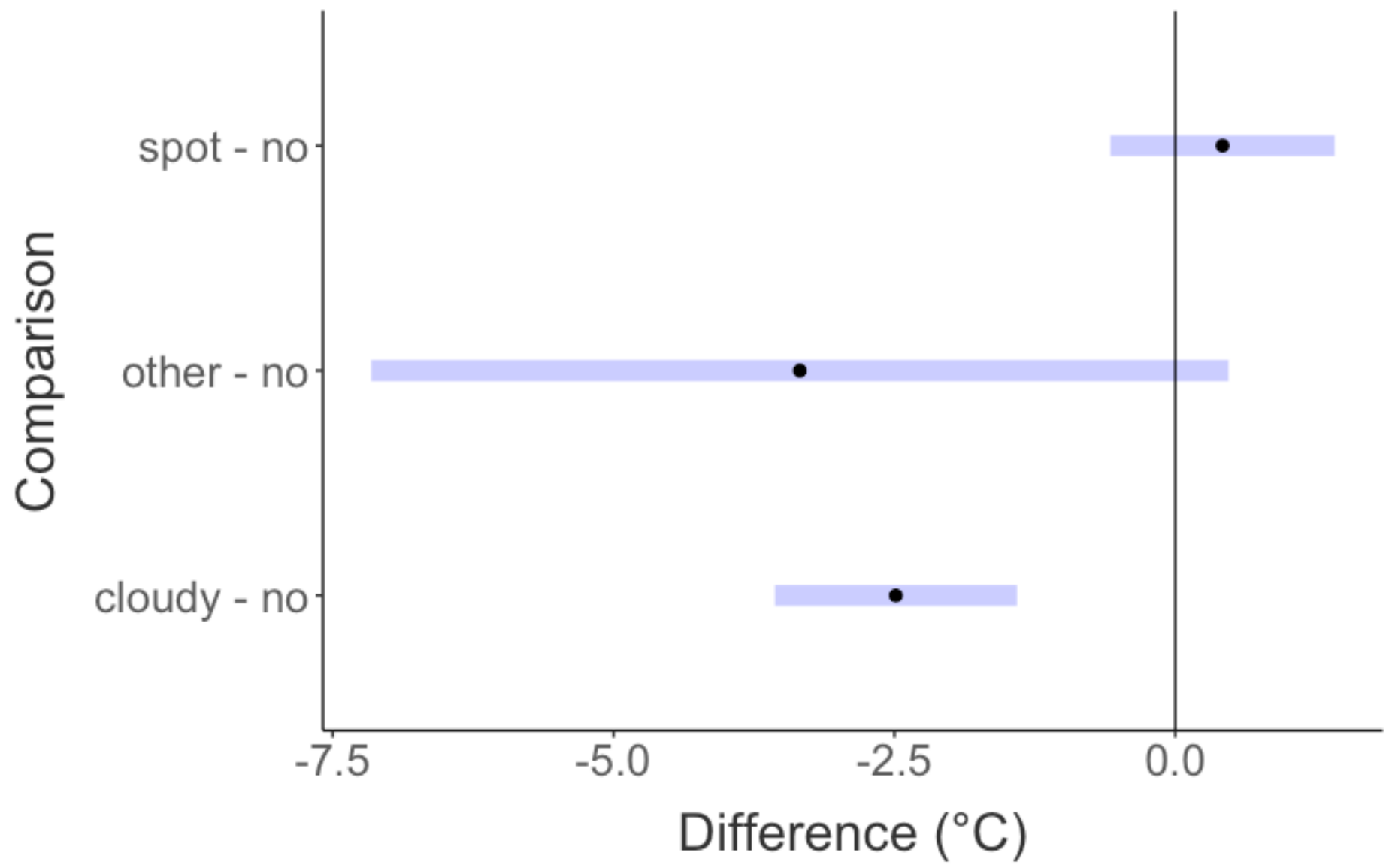

Figure S7 – Pathogen effect sizes (i.e. potential effect of the pathogen on CTmax). Contrasts between individuals with and without pathogen phenotypes, based on the estimated marginal means from a mixed effects model examining CTmax as a function of species and collection temperature (with interaction), along with the pathogen phenotype.

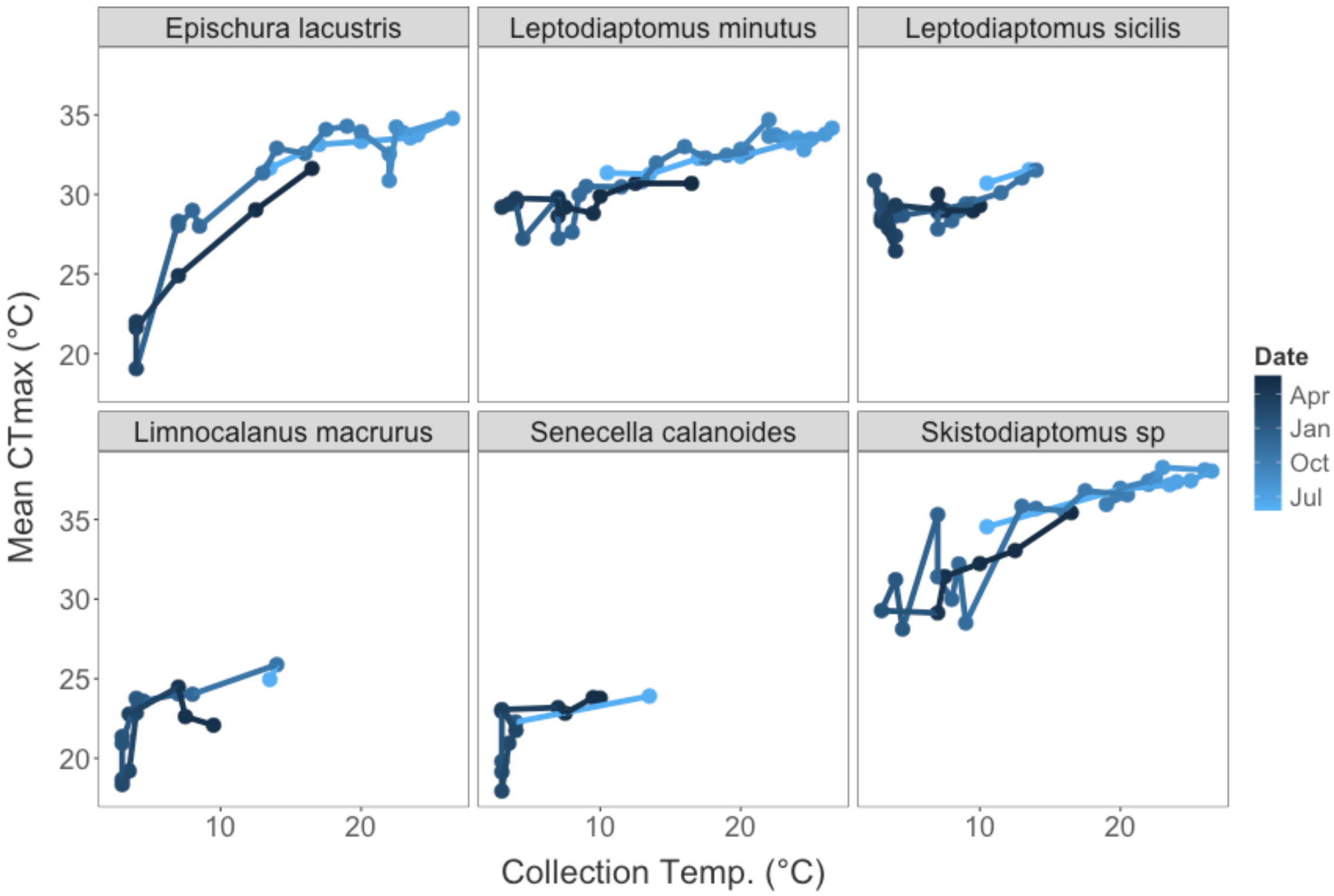

Figure S8 – The relationship between collection temperature and mean CTmax. Points are connected in chronological order, highlighting the temporal change in thermal limits. Earlier samples are shown in darker colors, with later samples shown in progressively lighter shades. The overlap of thermal limits during warming and cooling periods suggests changes are driven by acclimation (a reversible process), rather than processes like adaptation.

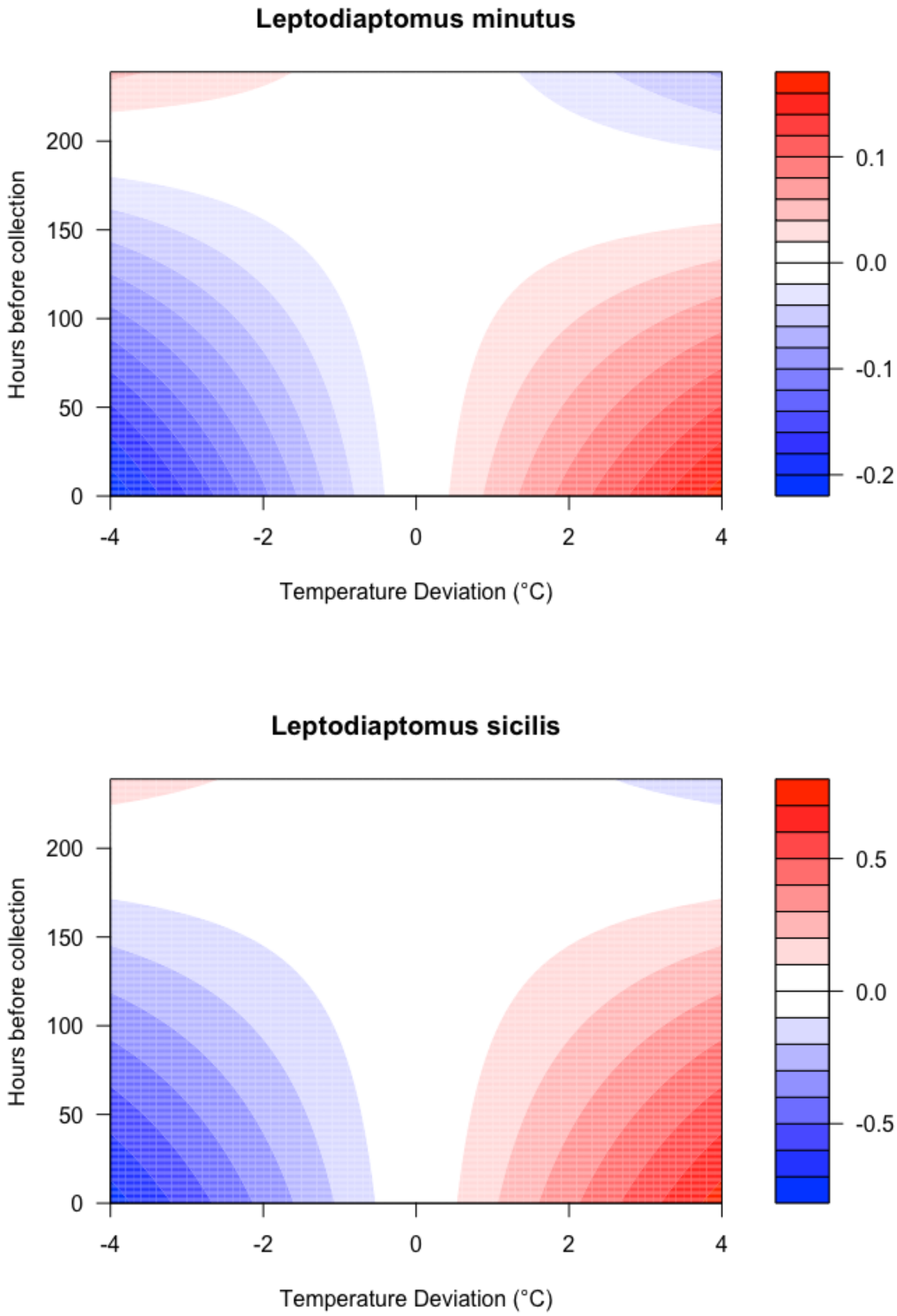

Figure S9 – Response surfaces from the distributed lag non-linear model showing the deviation of CTmax (color of the shading) as a result of temperature deviation (x-axis) and the time before collection (y-axis), for the two Leptodiaptomus species.

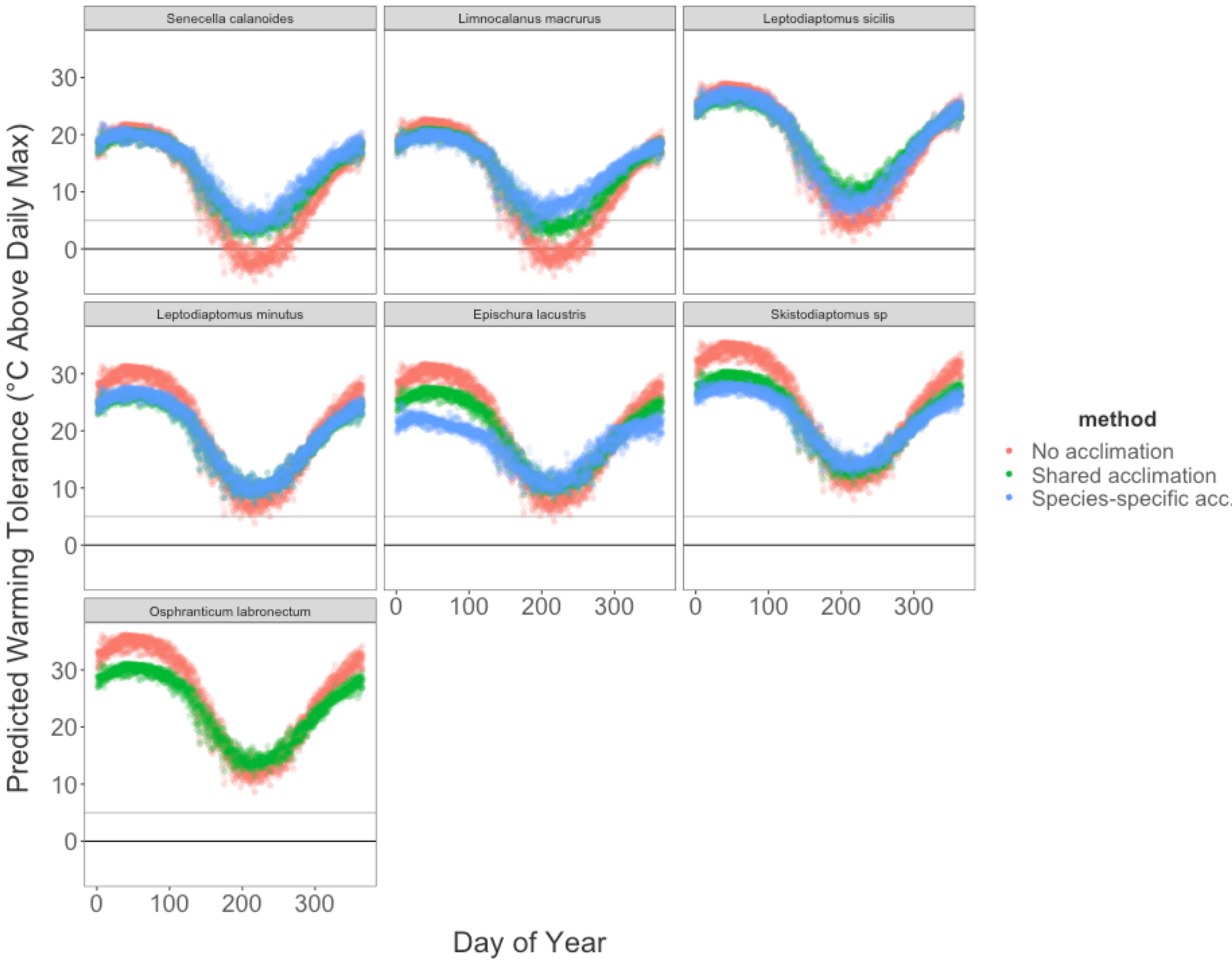

Figure S10 – Hindcasted warming tolerance estimates based on three acclimation scenarios. Estimated warming tolerance is shown against day of the year.

Table S1 – Formulas used to hindcast thermal limits for each species. Hindcast 1 uses the average thermal limit for each species. Hindcast 2 incorporates a shared acclimation capacity across all species (0.2907 x the mean daily temperature). Hindcast 3 allows both acclimation capacity and the cue to vary across species.

**Species**	**Hindcast 1**	**Hindcast 2**	**Hindcast 3**
Senecella calanoides	22.04	21 + 0.2907 x temperature	20.61 + 0.33 x nineteen-day max
Limnocalanus macrurus	22.89	21.26 + 0.2907 x temperature	20.97 + 0.48 x eight-day min
Leptodiaptomus sicilis	29.16	27.37 + 0.2907 x temperature	28.26 + 0.17 x mean temp
Leptodiaptomus minutus	31.54	27.19 + 0.2907 x temperature	27.69 + 0.27 x mean temp
Epischura lacustris	31.85	27.75 + 0.2907 x temperature	21.54 + 0.53 x twenty-nine-day max
Skistodiaptomus sp	35.67	30.53 + 0.2907 x temperature	28.32 + 0.41 x two-day max
